# Mechanical strain promotes osteoblast ECM formation and improves its osteoinductive potential

**DOI:** 10.1186/1475-925X-11-80

**Published:** 2012-10-25

**Authors:** Yong Guo, Chun-qiu Zhang, Qiang-cheng Zeng, Rui-xin Li, Lu Liu, Qin-xin Hao, Cai-hong Shi, Xi-zheng Zhang, Yu-xian Yan

**Affiliations:** 1 Academy of Military Medical Science, Tianjin Institute of Medical Equipment, No 106 Wandong Road, Hedong District, Tianjin, 300161, China; 2 School of Mechanical Engineering, Tianjin University of Technology, No. 263 Hongqi Nalu Road ,Nankai District, Tianjin, 300191, China; 3 Shandong provincial Key lab of biophysics, Dezhou University, No. 566 Daxuexi Road, Decheng District, Dezhou, 253021, China

**Keywords:** Tensile strain, Osteoblast, Extracellular matrix, Osteoinduction

## Abstract

**Background:**

The extracellular matrix (ECM) provides a supportive microenvironment for cells, which is suitable as a tissue engineering scaffold. Mechanical stimulus plays a significant role in the fate of osteoblast, suggesting that it regulates ECM formation. Therefore, we investigated the influence of mechanical stimulus on ECM formation and bioactivity.

**Methods:**

Mouse osteoblastic MC3T3-E1 cells were cultured in cell culture dishes and stimulated with mechanical tensile strain. After removing the cells, the ECMs coated on dishes were prepared. The ECM protein and calcium were assayed and MC3T3-E1 cells were re-seeded on the ECM-coated dishes to assess osteoinductive potential of the ECM.

**Results:**

The cyclic tensile strain increased collagen, bone morphogenetic protein 2 (BMP-2), BMP-4, and calcium levels in the ECM. Compared with the ECM produced by unstrained osteoblasts, those of mechanically stimulated osteoblasts promoted alkaline phosphatase activity, elevated BMP-2 and osteopontin levels and mRNA levels of runt-related transcriptional factor 2 (Runx2) and osteocalcin (OCN), and increased secreted calcium of the re-seeded MC3T3-E1 cells.

**Conclusion:**

Mechanical strain promoted ECM production of osteoblasts in vitro, increased BMP-2/4 levels, and improved osteoinductive potential of the ECM. This study provided a novel method to enhance bioactivity of bone ECM in vitro via mechanical strain to osteoblasts.

## Introduction

The extracellular matrix (ECM) is a non-cellular component of tissues and contains various protein fibers interwoven in a hydrated gel composed of a network of glycosaminoglycan chains that are secreted by resident cells to provide a mechanical support for cell growth, adhesion, proliferation, differentiation, morphology, and gene expression [1-3]. The ECM is a potent regulator of cell function and differentiation, and provides a supportive microenvironment for mammalian cells in vitro; therefore, it is a very suitable scaffold material for tissue engineering [4,5]. The ECM produced by osteoblasts is the major component of mature bone and mechanical strain plays an important role in growth and development of osteoblasts and bone tissue [6,7]; hence, the investigation of mechanical stimuli affecting ECM formation, especially produced in vitro, presents a particularly promising line of research.

Osteoblasts are important mechanical receptors that can transform mechanical stimuli into biochemical signals for bone matrix formation and promote mineralization [8]. Mechanical strain promotes matrix mineralization of osteoblasts [8,9] and increases the expression of ECM-related proteins of osteoblasts, including osteonectin, osteopontin (OPN), osteocalcin (OCN), bone morphogenetic protein 2 (BMP-2), and type I collagen [10]. In addition, mechanical strain of osteoblasts promotes matrix-bound vascular endothelial growth factor (mVEGF) synthesis, which has angiogenic properties in vivo [11,12]. In these studies, most of ECM-related proteins were intracellular. Actually, the influence of mechanical stimuli on ECM formation in vitro is not fully understood and its in vitro effects on levels of collagen and BMPs in the ECM remain unexplored.

In recent years, a considerable effort has been put into in vitro research to investigate the bioactivity of osteoblastic ECM formation. In general, osteoblasts are cultured on cell culture plates or dishes and removed using chemical or physical methods and the ECM attached to the dishes is prepared. The bone-specific ECM produced by osteogenic cells (MC3T3-E1) promoted the differentiation of embryonic stem cells [13]. Our study demonstrated that the ECM of primary osteoblasts in vitro can promote differentiation of preosteoblasts [14]. So these studies are likely to contribute to ECM-modified biomaterial scaffold for bone cell/tissue engineering. However, the influence of mechanical strain on bioactivity of osteoblast ECM remains unexplored.

In the present study, we stimulated mouse osteoblastic MC3T3-E1 cells cultured in dishes with mechanical tensile strain, prepared the ECM-coated dishes, then assayed the ECM proteins and calcium and re-seeded MC3T3-E1 cells on ECM-coated dishes to assess the osteoinductive potential of the ECM. Also, we investigated the influence of mechanical strain on ECM formation and bioactivity in vitro, which provided a novel method to enhance ECM bioactivity via application of mechanical strain to osteoblasts.

## Materials and methods

### Preparation of osteoblast-derived ECM-coated cell culture dishes

MC3T3-E1 cells, a mouse monoclonal pre-osteoblastic cell line that has been shown to differentiate into osteoblasts and osteocytes [15,16], were maintained on mechanical loading dishes that were reformed from cell culture dishes (Nalge Nunc International, Roskilde, Denmark) in alpha minimal essential medium (α-MEM; Invitrogen, Carlsbad, CA, USA) supplemented with 10% fetal calf serum and 1% penicillin-streptomycin.

At confluence, the MC3T3-E1 cells were cultured in α-MEM medium containing 10 mM β-glycerophosphate and 250 μM ascorbic acid 2-phosphate, and subjected to mechanical tensile strain of 2500 microstrain (με) at 0.5 Hz for 1 h/day at indicated times. The mechanical strain was generated by a specially designed four-point bending device, as previously described [17-19]. The device was driven by a stepping motor (controlled by a single chip microcomputer) and has been shown to produce homogenous cell culture substrate that is composed predominantly of uniaxial cells with the same deformations [20,21].

**Figure 1:**
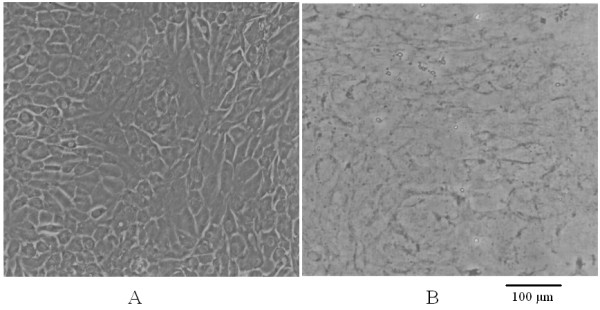
**Preparation of osteoblast ECM which was coated on dishes.** Osteoblasts were observed via inverted microscopy (**A**). After treatment with PBS containing 0.5% Triton X-100 and 0.10 M NH_4_OH, the cells were removed and the ECM attached to dishes was prepared (**B**).

The cells were removed according to an established method with some modifications [22]. Briefly, after washing with PBS, the cells were removed by incubation for 3 min with PBS containing 0.5% Triton X-100 and 10 mM NH_4_OH at room temperature then washed three times with PBS. The ECMs attached to the dishes were treated with 100 units/ml DNase (Sigma-Aldrich, St. Louis, MO, USA) for 1 h and the resulting ECMs were rinsed with PBS, observed by inverted microscopy ( Figure 1), then allowed to dry and stored at 4°C for further use. Unstrained cultures (control) were maintained under identical culture conditions without mechanical loading.

### Assay of ECM formation

A. Measure hydroxyproline content. The ECMs coating the dishes was hydrolyzed and the hydroxyproline content was detected with the Chloramine-T Hydroxyproline Assay Kit (Nanjing Jiancheng Biotechnology Co., Ltd., Nanjing, China) according to the manufacturer’s protocol.

B. Calcium deposition measurement. After the ECM-coated dishes were treated overnight with 0.1 M HCl, the ECM-deposited calcium content of the dishes was measured with the Calcium Assay Kit (Nanjing Jiancheng Biotechnology Co., Inc.) using the methyl thymol blue complexon method according to the manufacturer’s instructions.

C. Western blot analysis of BMP-2 and BMP-4 in ECMs. The ECMs were scraped off the dishes with a cell scraper and lysed by brief sonication on ice in Protein Extraction Reagent (Novagen; Merck KGaA, Darmstadt, Germany). The protein concentration of the lysates was measured according to bicinchoninic acid assay method. Briefly, equal amounts of protein were separated by sodium dodecyl sulfate polyacrylamide gel electrophoresis and electrotransferred onto polyvinylidene difluoride membranes (Millipore, Bedford, MA, USA). After blocking with 5% skim milk, the membranes were incubated overnight with the primary antibody at 4°C. After incubation with horseradish peroxidase-conjugated secondary antibody, the immunoreactive bands on the membranes were visualized using an enhanced chemiluminescence detection kit (Santa Cruz Biotechnology, Santa Cruz, CA, USA). The optical densities of the protein bands were determined with Gel Doc 2000 (Bio-Rad, Hercules, CA, USA). The expression of glyceraldehyde3-phosphatedehydrogenase (GAPDH) of MC3T3-E1 cells was used as a loading control substitute for total ECM proteins and data were normalized against those of corresponding GAPDH.

### Osteoblastic differentiation of re-seeded MC3T3-E1 cells on ECM-coated dishes

Cells were divided into three groups:

1) The cells that were seeded on dishes without ECM were indicated as the “no ECM” group.

2) The cells that were re-seeded on dishes coated with ECM produced by unstrained MC3T3-E1 cells were indicated as the “unstrain” group.

3) The cells that were re-seeded on dishes coated with ECM produced by mechanically strained MC3T3-E1 cells were indicated as the“strain” group.

A. Alkaline phosphatase (ALP) activity assay and calcium measurement. After trypsinization and centrifugation, the cells were lysed by brief sonication on ice in a lysis buffer (10 mM HEPES, 250 mM sucrose, 5 mM Tris–HCl, and 0.1% TritonX-100 at pH 7.5). The ALP activity of the cell lysates was assayed with an ALP activity assay kit (Nanjing Jiancheng Biotechnology Co. Ltd. Nanjing China) at 25°C using the p-nitrophenyl phosphate method according to manufacturer^’^s protocol. ALP activity of each sample was normalized to the protein concentration. After the cells on the ECM-coated dishes were removed, calcium deposition on the dishes was measured using a calcium assay kit, the calcium deposition content of the ECM on which no cells were re-seeded was subtracted, and the remnant calcium content was secreted by the cells.

B. Western blot analysis of intracellular BMP-2 and OPN. Cell lysates were harvested with radio immunoprecipitation lysis buffer (pH 7.4, 50 mM Tris, 150 mM NaCl, 1% NP-40, 0.5% sodium deoxycholate, 0.1% sodium dodecyl sulfate, and protease inhibitors in the buffer: 2 mM sodium pyrophosphate, 25 mM β-glycerophosphate, 1 mM ethylenediaminetetraacetic acid, 1 mM Na_3_VO_4,_ 0.5 μg/ml leupeptin, and 1 mM phenylmethylsulfonyl fluoride). The protein content of the cell lysates was quantified and then western blotting was performed according to the method mentioned above.

C. Real time polymerase chain reaction (PCR) analysis. Total RNA (1 μg) was extracted with Trizol reagent (Invitrogen) for cDNA synthesis using the Rever TraPlus Kit (Toyobo Co., Ltd., Osaka, Japan). Real-time PCR was performed to detect mRNA levels of runt-related transcriptional factor 2 (Runx2), OCN, and GAPDH (internal control reference) using SYBR Green I PCR Mix (Invitrogen) on an Real-Time PCR System (7900; Applied Biosystems, Foster City, CA, USA) according to the manufacturer’s instructions. Primer sequences are listed in Table 1. The amplification reaction included a denaturation step at 94°C for 3 min followed by 40 cycles of 94°C for 15 s, and annealing and extension at each annealing temperature for 30 s. Using the relative quantitative method (2^-ΔΔCt^), the expression levels of the PCR products of interest were calculated relative to those in the control group.

**Table 1:** Primers used for real-time PCR analysis

**Gene**	**Primer sequence (5’-3’)**	**length (bps)**
Runx2	F:AGTAGCCAGGTTCAACGAT	90
	R:GGAGGATTTGTGAAGACTGTT	
OCN	F:AGTCTGACAAAGCCTTCA	134
	R:AAGCAGGGTTAAGCTCACA	
GAPDH	F: ACCCATCACCATCTTCCAGGAG	159
	R: GAAGGGGCGGAGATGATGAC	

### Statistical analysis

The data are presented as means ± standard deviation and analyzed using SPSS v10.0 software (SPSS, Inc., Chicago, IL, USA) using one-way analysis of variance. A *P*-value < 0.05 was considered statistically significant.

## Results

### Mechanical strain elevates hydroxyproline and calcium content and increases BMP-2 and BMP-4 protein levels in osteoblast ECM

**Figure 2:**
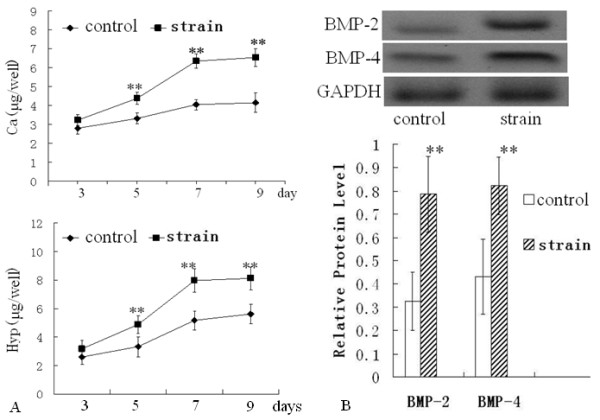
**Calcium, hydroxyproline, and BMPs levels in the ECM coated on dishes.** The mechanical strain increased the contents of calcium and hydroxyproline of the ECM at the indicated times (**A**), the mechanical stimulus for 7 days enhanced the relative protein expression levels of BMP-2 and BMP-4 (**B**). *P < 0.05, ** P < 0.01, between indicated groups. Quantitative results are the means ± standard error of 6 experiments.

After subjecting MC3T3-E1 cells to a mechanical tensile strain of 2500 με at 0.5 Hz for 1 h/day, the ECM hydroxyproline and calcium content, which was produced by the strained cells and attached to dishes, were both increased compared with those of ECM produced by non-stimulated cells (control group). Along with the duration of culturing, the hydroxyproline and calcium content were both significantly increased for 9 days (Figure 2A). Resulting hydroxyproline measurements were finally converted to collagen contents following a 1:10 (hydroxyproline:collagen) ratio [23]. Therefore, the change of hydroxyproline equated to the change of collagen content.

After exposing the cells to mechanical strain for 7 days, western blot analysis indicated that the BMP-2 and BMP-4 levels in the ECM were both greater than that in the control group (Figure 2B), suggesting that mechanical strain elevated the levels of collagen, calcium, and BMP-2/4 in osteoblast ECMs.

### The ECM produced by mechanically stimulated MC3T3-E1 cells enhanced ALP activity and increased BMP-2 and OPN protein levels, calcium deposition, and mRNA levels of Runx2 and OCN of re-seeded cells

**Figure 3:**
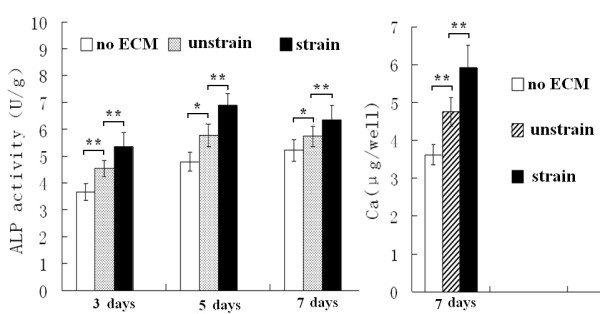
**ALP activity and calcium of MC3T3-E1 cells re-seeded on the different ECMs.** The ECM produced by the cells stimulated with mechanical strain (“strain” group) enhanced ALP activity and calcium content at the indicated times. * P < 0.05, ** P < 0.01, between indicated groups. Quantitative results are the means ± standard error of 6 experiments.

**Figure 4:**
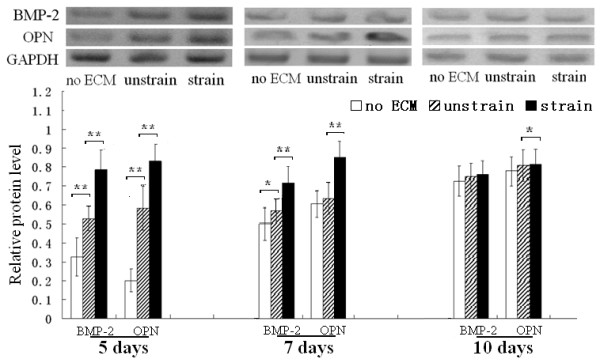
**BMP-2 and osteopontin protein levels of MC3T3-E1 cells re-seeded on different ECMs.** The ECM produced by the mechanically strained cells enhanced the relative protein expression levels of BMP-2 and OPN in cells cultured for 5 and 7 days (**A**/**B**). After 10 days of culture, the ECM had virtually no effect on the relative protein expression levels of BMP-2 and OPN (**C**). *P < 0.05, ** P < 0.01, between indicated groups. Quantitative results are the means ± standard error of 5 experiments.

After MC3T3-E1 cells were re-seeded on the dishes coated with the ECM, produced by the cells which were stimulated by mechanical strain for 7 days, ALP activity and calcium deposition of the MC3T3-E1 cells were both higher than the cells re-seeded on the ECM produced by the unstrained cells (unstrain group) (Figure 3). The western blot analysis of BMP-2 and OPN demonstrated that the cell protein levels were both higher than in the unstrain group after the re-seeded cells were cultured for 5 and 7 days (Figure 4A-B). After the re-seeded cells were cultured for 10 days, the levels of the 2 proteins were not enhanced compared with the unstrain groups (Figure 4C).

**Figure 5:**
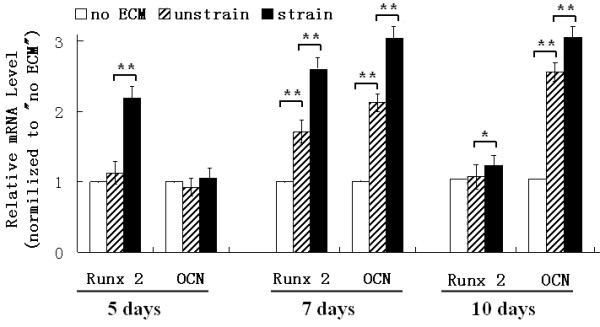
**Runx2 and OCN mRNA levels of MC3T3-E1 cells re-seeded on ECMs.** The ECM produced by the mechanically strained cells increased mRNA level of Runx2 after 5, 7, and 10 days of culture and increased OCN mRNA level after 7 and 10 days of culture. *P < 0.05, ** P < 0.01, between the indicated groups. Quantitative results are the means ± standard error of 7 experiments.

Additionally, the mRNA level of Runx2 was elevated after 5 and 7 days of culture, and OCN mRNA level was increased after 7 and 10 days of culture (Figure 5). Our results indicated that the ECM, produced by the mechanically stimulated cells, enhanced ALP activity, calcium deposition, BMP-2 and OPN protein levels, and mRNA levels of Runx2 and OCN.

## Discussion

Mechanical stimulus plays a significant role in growth and differentiation of osteoblasts. Previous studies indicated that mechanical strain increased matrix mineralization of osteoblasts derived from mesenchymal stem cells [8,9] and enhanced expression levels of bone ECM-related proteins/genes [10,24].

Our previous study demonstrated that mechanical tensile strain at a frequency of 0.5 Hz and intensities of 2000–3000 με for 1 h/day promoted osteoblast proliferation and differentiation (increased bone ECM proteins/genes such as collagen I, OCN, BMPs, etc.) [19,25,26] (some data not published), suggesting that mechanical tensile strain promoted osteoblast ECM formation. Therefore, in this study, we selected 0.5 Hz at 2500 με mechanical strain for 1 h/day.

However, to the best of our knowledge, the effect of mechanical strain on osteoblast ECM formation and its bioactivity or osteoinductive potential regarding ECM as a whole and independently has not been reported.

The ECM is secreted by resident tissue cells and is predominantly composed of structural proteins (i.e., collagen, fibronectin, laminin, etc.), glycosaminoglycans, and proteoglycans, as well as growth factors, chemokines, and cytokines [1,27]. ECM serves as a reservoir of growth factors and cytokines, such as BMP, fibroblast growth factor, and mVEGF, among others, which bind to either polysaccharide or protein constituents of the ECM [11,28,29] to regulate cell proliferation and differentiation.

In this study, we found that the ECM produced by mechanically stimulated MC3T3-E1 cells and attached on dishes contained more collagen, calcium, and BMP-2/4 than those produced by unstrained cells.

Collagen is the main component of ECMs and extracellular-deposited calcium is indicative of mineralized matrix production of osteoblasts. BMPs are members of the transforming growth factor superfamily and potent osteoblastic differentiation factors that play pivotal regulatory roles in bone formation [30,31]. They are purified from bone matrix and can induce transformation of mesenchymal stem cells into osteoblasts and chondrocytes, such as BMP-2 which induces ALP and OCN expression [32], and is capable of producing bone [33-35]. The BMP-4 amino sequence shares 83% homology with that of BMP-2 and also has the ability to promote osteogenesis [36,37]. The results of our study confirm the possibility that mechanical strain can promote ECM formation and increase BMPs (cytokines with osteoinductive potential) in ECMs produced by osteoblasts in vitro.

ECM deposited in vitro can induce osteoblastic differentiation of human mesenchymal stem cells and murine embryonic stem cells [13,38]. In our study, ECM-coated cell culture dishes were prepared. Compared with the ECM of unstrained osteoblastic cells, the ECM of mechanically strained cells promoted ALP activity, increased levels of BMP-2 and OPN and mRNA levels of Runx2 and OCN, and increased extracellular-deposited calcium concentrations in re-seeded osteoblastic cells.

ALP is widely used as a marker of osteogenic differentiation by increasing enzymatic activity to an osteoblastic phenotype [39,40]. BMP-2 and calcium are markers of osteoblastic differentiation. OPN, a secreted ECM protein found in bone matrix, is also a maker of osteogenesis [41]. ALP and OPN are markers of early differentiation [42], while OCN is a late marker corresponding with matrix deposition and mineralization [43]. Runx2 is the most critical transcription factor that regulates osteoblast differentiation and bone formation in vitro and in vivo [44]. During osteoblastic differentiation, BMP-2 regulates OCN expression through Runx2-dependent ATF6 expression, which directly regulates OCN transcription [45]. Runx2 directs osteoblastic differentiation of pluripotent mesenchymal cells and triggers the expression of major bone matrix protein genes [46]. Therefore, these results indicate that mechanical strain improves osteoinductive potential of the osteoblast ECM.

Additionally, in this study, after culturing the re-seeded cells for 10 days, the relative protein levels of BMP-2 and OPN were not enhanced compared with the unstrained groups. The re-seeded cells produced enough ECM to promote osteoblastic differentiation themselves, so the results are acceptable.

## Conclusions

The 2500 με mechanical strain promoted formation of the osteoblast ECM, increased BMPs in the ECM, and enhanced osteoinductive potential of the ECM. This study provides a novel method to enhance bioactivity of ECM or ECM biomaterial via application of mechanical strain to osteoblasts. It is likely to contribute to ECM-modified biomaterial scaffold for tissue engineering in the future.

## Abbreviations

ECM: Extracellular matrix; BMP: Bone morphogenetic protein; Runx2: Runt-related transcriptional factor 2; OCN: Osteocalcin; mVEGF: Matrix-bound vascular endothelial growth factor; α-MEM: Alpha minimal essential medium; με: Microstrain; OPN: Osteopontin; GAPDH: Glyceraldehyde3-phosphatedehydrogenase; ALP: Alkaline phosphatase; and PCR: Polymerase chain reaction.

## Competing interests

All authors declare that no conflict of interest exists.

## Authors’ contributions

YG carried out ECM preparation, participated in the interpretation of the results, and drafted the manuscript. CZ participated in the interpretation of the results, revised the manuscript, and designed the four-point bending device. QZ performed real-time PCR and Western blotting. RL cultured the cells and assayed of ECM and calcium content. LL performed the ALP assay and participated in drafting the manuscript. QH performed the Western blotting with QZ and participated in cell culturing. CS manufactured the four-point bending device and manipulated the device. XZ conceived the study and revised the final version of the article with CZ. YY participated in drafting and revising the manuscript. All authors read and approved the final manuscript.
